# Editorial: Biology of myxobacteria

**DOI:** 10.3389/fmicb.2024.1450345

**Published:** 2024-07-05

**Authors:** Zhoukun Li, Honghui Zhu, David E. Whitworth, David C. Stevens

**Affiliations:** ^1^Department of Microbiology and College of Life Sciences, Nanjing Agricultural University, Nanjing, China; ^2^Guangdong Institute of Microbiology, Guangdong Academy of Science, Guangzhou, China; ^3^Department of Life Sciences, Aberystwyth University, Aberystwyth, United Kingdom; ^4^Department of BioMolecular Sciences, School of Pharmacy, University of Mississippi, Oxford, MS, United States

**Keywords:** developmental biology, predation biology, fruiting body, bacterial communication, biological control, soil ecosystems

Myxobacteria are fascinating, soil-dwelling organisms which exhibit several noteworthy biological features. Despite being “lowly” Gram-negative bacteria, cells within a myxobacterial colony are able to communicate with each other in a sophisticated fashion, orchestrating population-wide changes in motility and gene expression. Myxobacteria are micropredators and can acquire nutrients by killing and consuming a broad range of prey organisms, including other bacteria, fungi and oomycetes. They have a profound influence on the composition of microbial communities and are considered apex bacterial predators and keystone taxa in soil ecosystems. When prey nutrients are scarce, cells of a sufficiently large myxobacterial population aggregate together to form multicellular fruiting bodies, within which a complex regulatory network causes cells to differentiate into spores. To achieve such coordinated motion, myxobacteria possess two mechanistically distinct motors for swarming motility. They are also prolific producers of secondary metabolites, many of which have useful biological activities. Myxobacterial natural products have seen use in the clinic, and intact organisms are now also being used to prey upon pathogens, acting as biological control agents for protecting crops.

Over the past few decades, myxobacteria have primarily been investigated as model organisms, providing fundamental insights into specific biological processes such as motility and development. With time, researchers have increasingly investigated how those processes overlap and inter-depend on each other—for instance, motility is a pre-requisite for predation and multicellular development, while secondary metabolite production is required for predation. Fundamental research has also increasingly overlapped with applied research into potential exploitation of myxobacterial behaviors and biomolecules. The intention of this Research Topic was to bring together research articles and reviews spanning the breadth of myxobacterial biology, with special emphasis placed on the application of myxobacteria and their products as biological resources, as well as the overlap between developmental biology, predation biology, and myxobacterial ecology.

The Research Topic has successfully drawn together 14 submissions, including 12 Original Research articles and two Reviews. Some authors have focused on providing fundamental insights into specific aspects of myxobacterial biology, while others have addressed broader Research Topics which draw on overlapping subject areas. Several articles demonstrate how fundamental knowledge can be leveraged to inform potential applications to benefit humankind, while the two Review articles provide valuable contextualization of important myxobacterial behaviors.

Multicellular development is arguably the most distinctive feature of myxobacteria biology. A key regulator of development is the MrpC DNA-binding protein, which sequentially triggers distinct aspects of the developmental process at different levels of expression. Induction of *mrpC* expression is complicated, involving several regulatory proteins/pathways, while MrpC also acts as a negative autoregulator, down-regulating its own expression. Negative autoregulation (NAR) is a mode of feedback which typically suppresses transcriptional noise during gene expression. In their Original Research article, McLaughlin and Higgs investigated MrpC NAR by disrupting the MrpC-binding sites in the *mrpC* promoter region, which caused cells to lose their synchronicity, with subsets of cells swarming out of nascent cellular aggregates as “developmental swarms” rather than completing the developmental programme. Myxobacterial cells are rod-shaped and move along their long axes, periodically reversing their direction of travel (reversals are suppressed during development). Using mathematical modeling of experimental data, Chen et al. propose in their Original Research article that reversal frequency is affected by mechanical cues (such as the stiffness of the substrate over which the cell is moving, or cell-cell contact), which are directly and indirectly sensed by the motility machineries of the cell. Testable hypotheses are proposed by the authors, which will be invaluable in further deciphering the molecular mechanisms underlying the regulation of motility.

An increasingly studied myxobacterial behavior is their predation of other microbes. The Review article by Contreras-Moreno et al. provides a wonderful summary of a rapidly growing body of literature. Focusing on *Myxococcus xanthus*, the authors explain the importance of synergism between motility and the various (diverse) mechanisms of killing prey organisms, which include contact-dependent intoxification of prey, and the generalized secretion of toxic enzymes and chemicals, including redox-active metals. Predation can be resisted by prey organisms, and Müller et al. have shown in their Original Research article that the production of thiocillins by strains of *Bacillus cereus* helps them resist predation by *M. xanthus* and even fight back. The secretion of specialized antimicrobial metabolites by both “predator” and “prey” is clearly a key determinant of the outcome of microbial competition in the soil. Another determinant of predatory success is the production of flagella by prey. In their Original Research article, Zhang N. et al. have shown that a non-flagellated mutant of *Escherichia coli* is relatively resistant to predation by *M. xanthus* and that an *E. coli* dihydrouridine synthase B, *dusB*, mutant is more resistant to the *M. xanthus* antibiotic myxovirescin. Such studies also allude to the likely uniqueness of predator-prey interactions between different species/strains.

The Original Research article by Han et al. showcases the potential for harnessing myxobacterial predatory activity by applying them as crop protection agents. In their study they showed a strain of *Myxococcus fulvus* (and its protein secretions) could kill the causative agent of fireblight (*Erwinia amylovora*) *in vitro*, and could protect pear explants and seedlings from disease. The article eloquently supports the premise of the Review article by Zhang L. et al. in which they highlight the activity of myxobacteria against plant pathogens. The molecular mechanisms myxobacteria employ to kill prey organisms are summarized, and the article anticipates increasing applications for myxobacteria in the context of biological control, particularly in crop protection. Future applications of myxobacteria in the field will need to consider the impact of myxobacteria on natural microbial communities. The Original Research article by Yang et al. assessed the *in vitro* predatory activity of a myxobacterium against >60 typical soil bacteria. They also investigated the impact of this myxobacterium on the diversity of a soil microcosm, finding that the myxobacterium drove large shifts in population structure. Nevertheless, some organisms (e.g., *Streptomyces* spp.) were relatively unaffected by myxobacterial addition, and some potential “prey” were able to coexist stably with the myxobacterium.

In addition to the Review by Zhang L. et al., several Original Research articles have focused on the production of hydrolytic exoenzymes by myxobacteria. In their study, Zhou X. et al. investigated glycoside hydrolases of *Corallococcus silvisoli* using differential proteomics of the secreted proteomes of cells grown in the presence/absence of cellulose and chitin. Phylogenetic analysis of differentially expressed GH19 family glycoside hydrolases identified features which may confer substrate specificity on the enzymes. Similarly, but starting with a genomic approach, the study by Yuan et al. identified predicted lipolytic enzymes in the genomes of 13 *Sorangium cellulosum* strains. A novel carboxylesterase, LipB, was characterized experimentally, and shown to have activity against a variety of ester substrates, including the antibiotic nitrocefin. Another novel lipase was shown by Zhou Y. et al. to be able to kill pathogenic prey organisms such as *E. coli* and *Staphylococcus aureus*. The ArEstA lipase was originally identified in the genome of a novel species of myxobacterium, *Archangium lipolyticum*, which was isolated from pig farm soil. Expression and purification of the lipase showed it was able to lyse drug-resistant *E. coli*, as was the parental organism.

Novel myxobacterial species are also described in two further articles. The Original Research article by Zang et al. describes how they discovered a novel species, *Hyalangium ruber*, in soil from a wetland park. The isolate was found to produce a variety of secondary metabolites, one of which exhibited moderate cytotoxicity against human cell lines—an uncommon but potentially useful activity exhibited by some myxobacteria. Using rhizospheric soil samples, Ahearne et al. were able to isolate type strains for nine novel myxobacterial species from seven different genera (*Archangium, Myxococcus, Nannocystis, Polyangium, Pyxidicoccus, Sorangium*, and *Stigmatella*). Genome sequence analysis of the novel strains allowed the authors to identify large biosynthetic gene clusters (BGCs) which typically encode enzymes for the production of secondary metabolites. The observation of clusters of hybrid BGCs in the genomes led to the proposal that proximal BGCs contribute to the metabolic diversity and adaptability of myxobacteria by promoting duplication and deletion of homologous modules between BGCs. In their Original Research article, Liu et al. isolated a novel halotolerant *Myxococcus* sp. strain MxC21 from forest soil. Genome sequencing revealed that the strain carried a plasmid, which is very unusual—MxC21 is only the second myxobacterial strain ever found to have contained a plasmid, and such plasmids can be extremely useful in expanding the genetic toolbox available to myxobacteria researchers.

In conclusion, new studies such as those included in this Research Topic on *Biology of myxobacteria* continue to expand our understanding of myxobacterial ecology and evolution, while providing us with strains and natural products for application in diverse arenas of human endeavor.

## Author contributions

ZL: Writing – original draft, Writing – review & editing. HZ: Writing – original draft, Writing – review & editing. DW: Writing – original draft, Writing – review & editing. DS: Writing – original draft, Writing – review & editing.

